# 2,2-Dichloro-*N*-(3-nitro­phen­yl)acetamide

**DOI:** 10.1107/S160053680706881X

**Published:** 2008-01-09

**Authors:** B. Thimme Gowda, Sabine Foro, Hartmut Fuess

**Affiliations:** a Department of Chemistry, Mangalore University, Mangalagangotri 574 199, Mangalore, India; b Institute of Materials Science, Darmstadt University of Technology, Petersenstrasse 23, D-64287 Darmstadt, Germany

## Abstract

The conformation of the N—H bond in the structure of the title compound (3NPDCA), C_8_H_6_Cl_2_N_2_O_3_, is *anti* to the *meta*-nitro group, similar to that in the structures of 2-chloro-*N*-(3-nitro­phen­yl)acetamide (3NPCA) and 2,2,2-trichloro-*N*-(3-nitro­phen­yl)acetamide (3NPTCA), and the *meta*-chloro group in 2,2-dichloro-*N*-(3-chloro­phen­yl)acetamide (3CPDCA). The geometric parameters of 3NPDCA are similar to those of 2,2-dichloro-*N*-phenyl­acetamide, 3CPDCA, 3NPCA, 3NPTCA and other acetanilides. Inter­molecular N—H⋯O hydrogen bonds link the mol­ecules into chains running along the *b* axis.

## Related literature

For related literature, see: Gowda & Weiss (1994 ▶); Gowda *et al.* (2000 ▶, 2006 ▶, 2007 ▶).
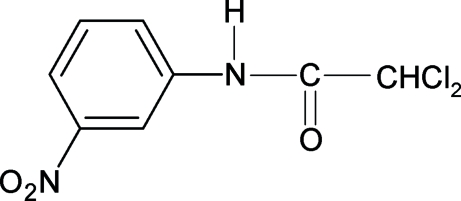


## Experimental

### 

#### Crystal data

C_8_H_6_Cl_2_N_2_O_3_
*M* *_r_* = 249.05
Orthorhombic, 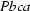

*a* = 9.6092 (6) Å
*b* = 10.6487 (7) Å
*c* = 19.868 (1) Å
*V* = 2033.0 (2) Å^3^
*Z* = 8
Mo *K*α radiation
μ = 0.63 mm^−1^
*T* = 299 (2) K
0.60 × 0.52 × 0.24 mm

#### Data collection

Oxford Diffraction Xcalibur diffractometer with Sapphire CCD Detector
Absorption correction: multi-scan (*SCALE3 ABSPACK*; Oxford Diffraction, 2007 ▶) *T*_min_ = 0.706, *T*_max_ = 0.865
11267 measured reflections
2072 independent reflections
1614 reflections with *I* > 2σ(*I*)
*R*_int_ = 0.022

#### Refinement

*R*[*F* ^2^ > 2σ(*F* ^2^)] = 0.032
*wR*(*F* ^2^) = 0.098
*S* = 1.10
2072 reflections
155 parameters
1 restraint
H atoms treated by a mixture of independent and constrained refinement
Δρ_max_ = 0.35 e Å^−3^
Δρ_min_ = −0.36 e Å^−3^

### 

Data collection: *CrysAlis CCD* (Oxford Diffraction, 2004 ▶); cell refinement: *CrysAlis RED* (Oxford Diffraction, 2007 ▶); data reduction: *CrysAlis RED*; program(s) used to solve structure: *SHELXS97* (Sheldrick, 2008 ▶); program(s) used to refine structure: *SHELXL97* (Sheldrick, 2008 ▶); molecular graphics: *PLATON* (Spek, 2003 ▶); software used to prepare material for publication: *SHELXS97*.

## Supplementary Material

Crystal structure: contains datablocks I, global. DOI: 10.1107/S160053680706881X/dn2307sup1.cif

Structure factors: contains datablocks I. DOI: 10.1107/S160053680706881X/dn2307Isup2.hkl

Additional supplementary materials:  crystallographic information; 3D view; checkCIF report

## Figures and Tables

**Table 1:** Hydrogen-bond geometry (Å, °)

*D*—H⋯*A*	*D*—H	H⋯*A*	*D*⋯*A*	*D*—H⋯*A*
N1—H1N⋯O1^i^	0.833 (16)	2.081 (17)	2.907 (2)	171 (2)

Symmetry code: (i) 
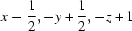
.

**Fig. 1.**
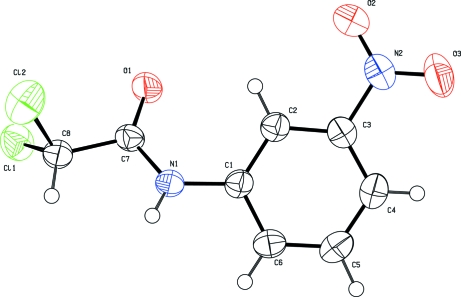
Molecular structure of the title compound showing the atom labelling scheme. Displacement ellipsoids are drawn at the 50% probability level. H atoms are represented as small spheres of arbitrary radii.

**Fig. 2.**
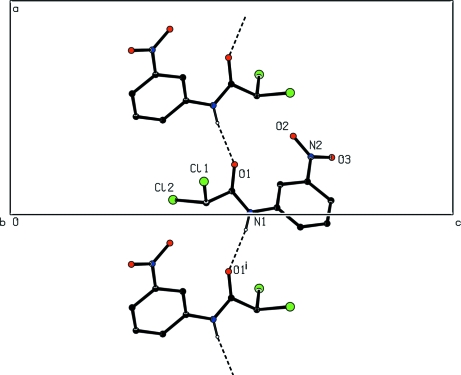
Partial packing view showing the hydrogen bonding as dashed lines.H atoms not involved in hydrogen bonding have been omitted for clarity. [Symmetry code: (i) x - 1/2, 1/2 - y,1 - z]

## supplementary crystallographic information

### Comment

As part of a study of the effect of ring and side chain substitutions on the solid state structures of acetanilides (Gowda *et al.*, 2000, 2006, 2007), in the present work, the crystal structure of 2,2-dichloro-*N*- (3-nitrophenyl)-acetamide (3NPDCA) has been determined to explore the effects of polar substituent groups on the structures of N-aromatic amides. The conformation of the N—H bond in the structure of 3NPDCA (Fig.1) is *anti* to the *meta* nitro group, similar to that in the structure of 2-chloro-*N*-(3-nitrophenyl)-acetamide (3NPCA) (Gowda *et al.*, 2007) and 2,2,2-trichloro-*N*-(3-nitrophenyl)-acetamide (3NPTCA)(Gowda *et al.*, 2000) and *meta* chloro group in 2,2-dichloro-*N*-(3-chlorophenyl)-acetamide (3CPDCA)(Gowda *et al.*, 2006). The geometric parameters in 3NPDCA are similar to those of 2,2-dichloro-*N*-(phenyl)-acetamide, 3CPDCA, 3NPCA, 3NPTCA and other acetanilides. The intermolecular N—H···O hydrogen bonds (Table 1) link the molecules into chains running along the *b* axis (Fig. 2).

### Experimental

The title compound was prepared similar to the literature method (Gowda and Weiss, 1994). The purity of the compound was checked by determining its melting point. It was characterized by recording its infrared and NQR spectra (Gowda and Weiss, 1994). Single crystals of the title compound were obtained from an ethanolic solution and used for X-ray diffraction studies at room temperature.

### Refinement

The H atoms were located in difference map with C—H = 0.89 (3)–0.96 (3) Å and N—H distance was restrained to 0.86 (2) %A. All H atoms were refined with isotropic displacement parameters (set to 1.2 times of the *U*_eq_ of the parent atom).

### Crystal data

**Table:**

C_8_H_6_Cl_2_N_2_O_3_	*F*_000_ = 1008
*M**_r_* = 249.05	*D*_x_ = 1.627 Mg m^−^^3^
Orthorhombic, *P**b**c**a*	Mo *K*α radiation λ = 0.71073 Å
Hall symbol: -P 2ac 2ab	Cell parameters from 4893 reflections
*a* = 9.6092 (6) Å	θ = 2.8–27.8º
*b* = 10.6487 (7) Å	µ = 0.63 mm^−^^1^
*c* = 19.868 (1) Å	*T* = 299 (2) K
*V* = 2033.0 (2) Å^3^	Prism, yellow
*Z* = 8	0.60 × 0.52 × 0.24 mm

### Data collection

**Table:**

Oxford Diffraction Xcalibur diffractometer with Sapphire CCD Detector	2072 independent reflections
Radiation source: fine-focus sealed tube	1614 reflections with *I* > 2σ(*I*)
Monochromator: graphite	*R*_int_ = 0.022
*T* = 299(2) K	θ_max_ = 26.4º
Rotation method data acquisition using ω and φ scans.	θ_min_ = 3.0º
Absorption correction: multi-scan(SCALE3 ABSPACK; Oxford Diffraction, 2007)	*h* = −12→11
*T*_min_ = 0.706, *T*_max_ = 0.865	*k* = −11→13
11267 measured reflections	*l* = −24→20

### Refinement

**Table:**

Refinement on *F*^2^	Secondary atom site location: difference Fourier map
Least-squares matrix: full	Hydrogen site location: inferred from neighbouring sites
*R*[*F*^2^ > 2σ(*F*^2^)] = 0.032	H atoms treated by a mixture of independent and constrained refinement
*wR*(*F*^2^) = 0.098	*w* = 1/[σ^2^(*F*_o_^2^) + (0.0385*P*)^2^ + 1.4567*P*] where *P* = (*F*_o_^2^ + 2*F*_c_^2^)/3
*S* = 1.10	(Δ/σ)_max_ = 0.044
2072 reflections	Δρ_max_ = 0.35 e Å^−^^3^
155 parameters	Δρ_min_ = −0.36 e Å^−^^3^
1 restraint	Extinction correction: SHELXL, Fc^*^=kFc[1+0.001xFc^2^λ^3^/sin(2θ)]^-1/4^
Primary atom site location: structure-invariant direct methods	Extinction coefficient: 0.0230 (13)

### Special details

**Table:**

Geometry. All e.s.d.'s (except the e.s.d. in the dihedral angle between two l.s. planes) are estimated using the full covariance matrix. The cell e.s.d.'s are taken into account individually in the estimation of e.s.d.'s in distances, angles and torsion angles; correlations between e.s.d.'s in cell parameters are only used when they are defined by crystal symmetry. An approximate (isotropic) treatment of cell e.s.d.'s is used for estimating e.s.d.'s involving l.s. planes.
Refinement. Refinement of *F*^2^ against ALL reflections. The weighted *R*-factor *wR* and goodness of fit *S* are based on *F*^2^, conventional *R*-factors *R* are based on *F*, with *F* set to zero for negative *F*^2^. The threshold expression of *F*^2^ > σ(*F*^2^) is used only for calculating *R*-factors(gt) *etc*. and is not relevant to the choice of reflections for refinement. *R*-factors based on *F*^2^ are statistically about twice as large as those based on *F*, and *R*- factors based on ALL data will be even larger.

### Fractional atomic coordinates and isotropic or equivalent isotropic displacement parameters (Å^2^)

**Table:**

	*x*	*y*	*z*	*U*_iso_*/*U*_eq_	
C1	0.03253 (19)	0.14098 (18)	0.60262 (10)	0.0344 (4)	
C2	0.1408 (2)	0.05696 (19)	0.61012 (11)	0.0365 (4)	
H2	0.204 (2)	0.043 (2)	0.5757 (12)	0.044*	
C3	0.1543 (2)	−0.00244 (19)	0.67144 (11)	0.0380 (5)	
C4	0.0653 (2)	0.0166 (2)	0.72466 (11)	0.0453 (5)	
H4	0.075 (3)	−0.025 (2)	0.7632 (14)	0.054*	
C5	−0.0418 (2)	0.1009 (2)	0.71576 (12)	0.0490 (6)	
H5	−0.103 (3)	0.122 (2)	0.7521 (14)	0.059*	
C6	−0.0584 (2)	0.1632 (2)	0.65542 (11)	0.0426 (5)	
H6	−0.131 (3)	0.220 (2)	0.6503 (13)	0.051*	
C7	0.1102 (2)	0.24984 (19)	0.50064 (11)	0.0357 (4)	
C8	0.0552 (2)	0.3297 (2)	0.44287 (11)	0.0398 (5)	
H8	−0.034 (3)	0.352 (2)	0.4488 (11)	0.048*	
N1	0.01034 (17)	0.20745 (17)	0.54149 (9)	0.0381 (4)	
H1N	−0.0711 (18)	0.227 (2)	0.5320 (12)	0.046*	
N2	0.2700 (2)	−0.09083 (18)	0.67926 (10)	0.0491 (5)	
O1	0.23372 (15)	0.22854 (16)	0.50684 (9)	0.0532 (5)	
O2	0.3669 (2)	−0.08426 (19)	0.63930 (10)	0.0664 (5)	
O3	0.2672 (2)	−0.16436 (19)	0.72637 (10)	0.0759 (6)	
Cl1	0.15452 (6)	0.46874 (5)	0.43763 (3)	0.0529 (2)	
Cl2	0.06927 (8)	0.24208 (7)	0.36778 (3)	0.0661 (2)	

### Atomic displacement parameters (Å^2^)

**Table:**

	*U*^11^	*U*^22^	*U*^33^	*U*^12^	*U*^13^	*U*^23^
C1	0.0270 (9)	0.0403 (10)	0.0360 (10)	−0.0056 (8)	−0.0003 (8)	0.0025 (8)
C2	0.0334 (10)	0.0388 (10)	0.0374 (10)	−0.0034 (8)	−0.0001 (8)	0.0008 (8)
C3	0.0363 (10)	0.0346 (10)	0.0430 (11)	−0.0033 (8)	−0.0046 (9)	0.0017 (8)
C4	0.0494 (13)	0.0483 (12)	0.0382 (11)	−0.0071 (10)	−0.0014 (10)	0.0065 (10)
C5	0.0460 (13)	0.0583 (14)	0.0428 (12)	−0.0043 (11)	0.0103 (10)	−0.0006 (10)
C6	0.0311 (10)	0.0487 (12)	0.0479 (12)	−0.0012 (9)	0.0036 (9)	0.0014 (10)
C7	0.0272 (9)	0.0389 (10)	0.0409 (10)	0.0013 (8)	−0.0009 (8)	0.0020 (8)
C8	0.0307 (10)	0.0479 (12)	0.0409 (11)	0.0006 (9)	−0.0001 (9)	0.0045 (9)
N1	0.0239 (8)	0.0503 (10)	0.0401 (9)	0.0018 (7)	−0.0002 (7)	0.0076 (8)
N2	0.0507 (11)	0.0446 (10)	0.0521 (11)	0.0028 (9)	−0.0091 (10)	0.0056 (9)
O1	0.0258 (7)	0.0698 (11)	0.0641 (10)	0.0050 (7)	0.0044 (7)	0.0272 (8)
O2	0.0606 (11)	0.0718 (12)	0.0668 (12)	0.0250 (10)	0.0086 (10)	0.0109 (10)
O3	0.0786 (13)	0.0713 (12)	0.0778 (13)	0.0122 (11)	−0.0056 (11)	0.0361 (11)
Cl1	0.0558 (4)	0.0395 (3)	0.0635 (4)	−0.0031 (2)	−0.0001 (3)	0.0064 (2)
Cl2	0.0740 (5)	0.0778 (5)	0.0467 (4)	−0.0200 (4)	−0.0084 (3)	−0.0116 (3)

### Geometric parameters (Å, °)

**Table:**

C1—C2	1.380 (3)	C6—H6	0.93 (3)
C1—C6	1.386 (3)	C7—O1	1.215 (2)
C1—N1	1.422 (2)	C7—N1	1.335 (3)
C2—C3	1.379 (3)	C7—C8	1.523 (3)
C2—H2	0.93 (2)	C8—Cl1	1.764 (2)
C3—C4	1.376 (3)	C8—Cl2	1.765 (2)
C3—N2	1.464 (3)	C8—H8	0.90 (3)
C4—C5	1.377 (3)	N1—H1N	0.833 (16)
C4—H4	0.89 (3)	N2—O3	1.220 (3)
C5—C6	1.380 (3)	N2—O2	1.226 (3)
C5—H5	0.96 (3)		
			
C2—C1—C6	120.30 (19)	C1—C6—H6	120.0 (16)
C2—C1—N1	121.86 (17)	O1—C7—N1	125.26 (19)
C6—C1—N1	117.83 (18)	O1—C7—C8	121.32 (19)
C3—C2—C1	117.65 (19)	N1—C7—C8	113.42 (17)
C3—C2—H2	120.9 (15)	C7—C8—Cl1	108.99 (14)
C1—C2—H2	121.5 (15)	C7—C8—Cl2	108.35 (15)
C4—C3—C2	123.5 (2)	Cl1—C8—Cl2	110.62 (12)
C4—C3—N2	119.03 (19)	C7—C8—H8	112.2 (15)
C2—C3—N2	117.42 (19)	Cl1—C8—H8	107.9 (16)
C3—C4—C5	117.6 (2)	Cl2—C8—H8	108.8 (15)
C3—C4—H4	121.6 (17)	C7—N1—C1	125.44 (17)
C5—C4—H4	120.8 (17)	C7—N1—H1N	116.8 (17)
C4—C5—C6	120.8 (2)	C1—N1—H1N	117.5 (17)
C4—C5—H5	120.9 (16)	O3—N2—O2	123.4 (2)
C6—C5—H5	118.2 (16)	O3—N2—C3	118.5 (2)
C5—C6—C1	120.2 (2)	O2—N2—C3	118.10 (18)
C5—C6—H6	119.8 (16)		
			
C6—C1—C2—C3	−0.2 (3)	N1—C7—C8—Cl1	131.85 (17)
N1—C1—C2—C3	179.62 (18)	O1—C7—C8—Cl2	71.9 (2)
C1—C2—C3—C4	0.8 (3)	N1—C7—C8—Cl2	−107.71 (18)
C1—C2—C3—N2	−179.58 (18)	O1—C7—N1—C1	6.6 (4)
C2—C3—C4—C5	−0.8 (3)	C8—C7—N1—C1	−173.76 (18)
N2—C3—C4—C5	179.6 (2)	C2—C1—N1—C7	−35.6 (3)
C3—C4—C5—C6	0.1 (3)	C6—C1—N1—C7	144.2 (2)
C4—C5—C6—C1	0.5 (3)	C4—C3—N2—O3	15.6 (3)
C2—C1—C6—C5	−0.4 (3)	C2—C3—N2—O3	−164.0 (2)
N1—C1—C6—C5	179.8 (2)	C4—C3—N2—O2	−162.0 (2)
O1—C7—C8—Cl1	−48.5 (3)	C2—C3—N2—O2	18.3 (3)

### Hydrogen-bond geometry (Å, °)

**Table:**

*D*—H···*A*	*D*—H	H···*A*	*D*···*A*	*D*—H···*A*
N1—H1N···O1^i^	0.833 (16)	2.081 (17)	2.907 (2)	171 (2)

Symmetry codes: (i) *x*−1/2, −*y*+1/2, −*z*+1.
